# miR-1323 Promotes Cell Migration in Lung Adenocarcinoma by Targeting Cbl-b and Is an Early Prognostic Biomarker

**DOI:** 10.3389/fonc.2020.00181

**Published:** 2020-02-21

**Authors:** Huan Zhao, Chunlei Zheng, Yizhe Wang, Kezuo Hou, Xianghong Yang, Yang Cheng, Xiaofang Che, Shilin Xie, Shuo Wang, Tieqiong Zhang, Jian Kang, Yunpeng Liu, Dianzhu Pan, Xiujuan Qu, Xuejun Hu, Yibo Fan

**Affiliations:** ^1^Department of Respiratory and Infectious Disease of Geriatrics, The First Hospital of China Medical University, Shenyang, China; ^2^Department of Respiratory, The First Affiliated Hospital of Jinzhou Medical University, Jinzhou, China; ^3^Department of Medical Oncology, The First Hospital of China Medical University, Shenyang, China; ^4^Key Laboratory of Anticancer Drugs and Biotherapy of Liaoning Province, The First Hospital of China Medical University, Shenyang, China; ^5^Department of Pathology, Shengjing Hospital of China Medical University, Shenyang, China; ^6^Department of Pulmonary Medicine, The First Hospital of China Medical University, Shenyang, China

**Keywords:** lung adenocarcinoma, miR-1323, CBLB, prognosis, biomarker

## Abstract

**Purpose:** MicroRNAs are known to regulate cellular processes in non-small cell lung cancer (NSCLC) cells and predict prognosis. However, identification of specific microRNAs in NSCLC as potential therapeutic targets is controversial. We aim to determine the clinical significance of miR-1323 in the prognosis of patients with lung cancer and the potential mechanism.

**Patients and methods:** A bioinformatics approach was used to screen the importance microRNA in NSCLC through the online GEO database (GSE42425). The relationship between expression level of miR-1323 and overall survival of lung cancer patients was analyzed. Additionally, an independent corhort including 53 NSCLC cases that underwent resection validated the connection between miR-1323 and LUAD patients' overall survival. Next, the function of miR-1323 was studied *in vitro* by transient transfection. A more in-depth mechanism was studied through luciferase reporter gene experiments.

**Results:** High miR-1323 expression correlated with poor survival in NSCLC patients (*P* = 0.011), and in lung adenocarcinoma (LUAD) patients (*P* = 0.015) based on GEO database (GSE42425). In the independent cohort based on our hospital, high miR-1323 expression was associated with LUAD patients (*P* = 0.025). Moreover, transfection with mimics of miR-1323 showed an increased migratory capacity in LUAD A549 and HCC827 cells. In addition, E3 ubiquitin-protein ligase Casitas B-lineage Lymphoma-b (Cbl-b) was found to be the target genes of miR-1323 and significantly down regulated after mimics of miR-1323 transfection, and high Cbl-b expression predicted better prognosis in NSCLC and LUAD (*P* = 0.00072 and *P* = 0.02, respectively).

**Conclusion:** The miR-1323 promoted LUAD migration through inhibiting Cbl-b expression. High miR-1323 expression predicted poor prognosis in LUAD patients.

## Introduction

Non-small cell lung cancer (NSCLC) is a leading cause of cancer-related death and accounts for 80–85% of all lung cancer cases (1). The prognosis of patients with NSCLC is very poor and hence the majority of patients are diagnosed at an advanced stage owing to the lack of cancer-specific symptoms. Although epidermal growth factor receptor (EGFR) has emerged as a major target for NSCLC therapy, almost all patients on this treatment inevitably acquire drug resistance, but the prognosis of patients remains poor (2). Therefore, identification of new targets in lung cancer is urgently needed.

A previous study has shown that microRNAs are involved in multiple aspects of lung cancer such as cell proliferation, apoptosis, invasion, and EMT (3–6). Additionally, the survival of lung cancer patients has been predicted by a series of microRNAs such as miR-133b, miR-93-5p (7, 8). However, specific microRNAs playing a significant prognostic role in NSCLC are not determined.

Gene Expression Omnibus(GEO) Database (https://www.ncbi.nlm.nih.gov/gds/) is a gene expression database created and maintained by The National Center for Biotechnology Information (9). It contains high-throughput gene expression data submitted by research institutions around the world. Almost all gene expression detection data involved in the studies can be found in this database. Therefore, in this study, we used the online GEO Database to screen and determine the role of miR-1323 in predicting prognosis in lung cancer patients, which makes great significance for early screening and diagnosis. Previously study showed that knocking down of miR-1323 has been shown to restore radiosensitivity in radiation-resistance lung cancer cell (10). Besides, miR-1323 also suppressed the expression of PRKDC and enhanced DNA repair. However, whether miR-1323 is involved in regulating the prognosis of lung cancer patients is not known. We determined the expression of miR-1323 in lung cancer patients' samples from GEO datasets and frozen sections, and found that miR-1323 predicted poor survival in LUAD, whose mechanism might through promote the migration ability of lung cancer cells *in situ*. Furthermore, we found that the way for miR-1323 to regulate migration of LUAD was by targeting Cbl-b and high Cbl-b expression predicted better survival in NSCLC and LUAD patients.

## Materials and Methods

### Patients and Tissue Collection

Primary LUAD tissues (*n* = 53) were obtained from NSCLC patients with permission of Shengjing Hospital of China Medical University (Shenyang, China) between December 2009 and 2010. All the patients underwent the surgery and were histomorphology confirmed. The current study was examined and approved by the Research Ethics Committee of Shengjing Hospital of China Medical University.

### Bioinformatics Analysis

GEO Database was used to screen vital prognostic marker miRNA in early-stage lung adenocarcinoma. The search terms include following key words: [(“lung neoplasms” OR (“lung neoplasms” OR “lung cancer”)) AND [“mirnas” OR (“micrornas” OR “MicroRNA”)] AND (“gene expression” OR “expression”)] AND “stage.” Suitable gene set was selected for subsequent analysis.

GEO2R, which is a online tool provided by official, allows users to compare two or more groups of Samples in a GEO Series and identify genes that are differentially expressed across experimental conditions. We divided all samples into good prognosis groups and poor prognosis groups according to median survival time and use GEO2R to analysis differentially expressed genes between two groups.

The corresponding clinical data were also acquired from the GEO database. The expression value of miRNAs were collected for each case and then divided into miR-1323 high expression and miR-1323 low expression groups. Correlation of miR-1323 expression level and clinicopathologic parameters was evaluated by Spearman assay. Kaplan-Meier analysis were performed to do the survival analysis. The relationship between miR-1323 and clinical stges of LUAD patients was analyzed by OncomiR database (11). MicroRNA target predictions were performed by online databases based on different prediction methods, including miRDB (12), miRWalk (13), Targetscan (14), starBase (15). The annotation of predicted gene symbols was done by DAVID Bioinformatics Resources 6.7 (16), Kaplan- Meier Plotter (17) was used to screen the target mRNA related with prognosis of LUAD.

### RNA Isolation and Quantitative Real-Time PCR

Total RNA was purifed from formalin-fixed, paraffin-embedded tissue sections using miRNeasy FFPE Kit (Qiagen, USA) in accordance with manufacturer's protocol. RNA was quantified and purified at absorbance of 260/280 nm using NanoDrop spectrophotometer. cDNA was synthesized using the One Step PrimeScript^@^ miRNA (Takara, Naha, Japan). miR-1323 quantifcation was done using SYBR^@^ Premix Ex TaqTM II (Takara) Kit. qRT-PCR experiment was conducted in triplicates, normalized to U6 Small nuclear RNA and performed on the Applied Biosystems 7500 Thermocycler. Relative expression were calculated based on 2-ΔΔCt method.

### Cells and Cell Culture

The human lung adenocarcinoma cell lines (A549 and HCC827) were obtained from ATCC. The cells were cultured in an humidified atmosphere with 95%air and 5%CO_2_ at 37°C,and supplemented with RPMI-1640 medium (Gibco, ThermoFisher, Shanghai, China) with 10% heat-inactivated fetal bovine serum, streptomycin (100 U/mL) and penicillin (100 U/mL). A549 and HCC827 cells were split every 2–3 days at a concentration of 1.5^*^105 and cells/ml.

### Transient Transfection

Before Wound healing assays, Transwell assays, WB assays, ELISA and Dual luciferase reporter assays, cells were transfected with plasmids, miR-1323 mimics, or Cbl-b siRNAs at indicated doses and times using jetPRIME® (Polyplus Transfection, New York, USA) following the manufacturer's instructions. qRT-PCR and Western blot was performed to verify the transfection effciency.

### Western Blot

Protein expression were assessed through Western blot analysis. Cell lysates were obtained using RIPA lysis [0.1%SDS, 1%Triton-100, 1 mmol EDTA (pH 8.0), 150 mmol/L NaCl, 10 mmol/L Tris-HCl (pH 7.5)] supplemented with protease inhibitors (100 μg/ml PMSF and 2 μg/ml Aprotitin). The protein level was measured by Coomassie brilliant blue method. Total protein extract was separated by SDS-PAGE and electrophoretically transferred onto a PVDF membrane. After blocking with 5% non-fat milk in TTBS buffer, membranes were incubated with antibodies [β-actin (1:1000, sc-47778, Santa Cruz Biotechnology), Cbl-b (1:250, sc-8006, Santa Cruz Biotechnology), Cyclin E (1:1000, sc-377100, Santa Cruz Biotechnology), Occludin (1:1000, 331500, Life Technologies)] overnight at 4°C. Membranes were then incubated for 30 min with secondary antibodies. Protein was detected using an ECL reagent in the Electrophoresis Gel Imaging analysis system.

### Transwell

Transwell migration assays were performed to measure cell migratory ability. 1.0 × 10^4^ cells were seeded in the top chamber of 24-well transwell plates, Medium supplemented with serum in the lower chamber was used to attract the cells in the top chamber which were suspended in medium without serum or growth factors. After migration at 37°C for 24 h, the non-migratory cells were removed by cotton wool. The migratory cells on the membrane were stained with 0.1% Giemsa stain solution for 2 h and the cells were quantified by counting the number of cells in randomly chosen 4 fields under microscope at 200-fold magnification.

### Wound Healing Assay

Migration of A549 and HCC827 cells was investigated by wound healing assay. Viable cells were plated at 3 × 10^5^ cells per well in six-well culture plates using growth media containing 10% FBS. After the cells were attached, cells were transfected with miR-1323 mimic or Cbl-b siRNAs for 48 h. As the cells reached semi-confluence, *in vitro* scratch wounds were created by scraping the cell monolayers with a 1 ml sterile pipette tip. After washing away suspended cells, the cells were treated with normal growth media containing lower concentration FBS, in order to eliminate the effect of proliferation on results. Photomicrograph was taken immediately (time 0 h) with an inverted microscope equipped with a digital camera, and the wounded cultures were allowed to grow for 24 h at 37°C. At this time, another photomicrograph was taken at the same position. Photos were randomly selected per hole for comparative analysis. Migration was quantified by counting cell numbers at the indicated distances from the wound edge. Data shown are representative of minimum three independent experiments.

### ELISA

After treated A549 cells by transient transfection for 24 or 48 h, the culture supernatants were collected and the number of cells was counted. The levels of interleukin-6 (IL-6) were analyzed by human IL-6 ELISA kit (Biolegend, San Diego,USA.) according to the manufacturer's protocol and the optical density (OD) was measured at 450 nm with a microplate reader. Finally, IL-6 level was adjusted by the total number of cells.

### Dual Luciferase Reporter Assay

Dual luciferase reporter assay was performed according to our previously study (18). We initially obtained the 3′-UTR sequence of Cbl-b was obtained through gene synthesis (OriGene, Rockville, MD, USA), and cloned into the vector pMirTarget through two restriction enzyme cutting sites (SgfI-MluI), resulting in the generation of SC209114. All reagents and methods are provided by OriGene Technologies (OriGene, Rockville, MD, USA). The sequencing results were compared with the standard template sequences of the BLAST software on the PUBMED and CHROMAS software to identify the gene mutation loci. In order to generate the Cbl-b mutant reporter, the seed region was mutated to remove all complementary nucleotides to miR-1323. A549 cells were co-transfected with firefly luciferase reporter plasmids (0.5 μg), pRL-TK luciferase control vector (0.005 μg) and miR-1323 or NC (50 nmol) in the 24-well plates. Luciferase assays were performed 24 h after transfection, using the dual-luciferase reporter assay system (Promega, Madison, WI, USA) according to the manufacturer's protocol (18).

### Statistical Analysis and Graphing

Kaplan-Meier was performed to do survival analysis. X-tile program was used to choose the optimal cutoff value. Sperarman correlation analysis and Student's *t*-test and was used to analyze the comparisons between two miR-1323 expression levels and clinicopathological characteristics of patients. *P* < 0.05 was considered to be statistically significant. Statistical analyses were performed using SPSS 18.0 software (SPSS, Inc., Chicago, IL) and GraphPad Prism 7 (GraphPad, La Jolla, CA) were used for statistical analysis.

## Results

### miR-1323 Was High Expression in Early NSCLC With Poor Prognosis

Previous studies on NSCLC implied the prognostic predictive value of microRNAs, and microRNA targeting showed great potential as a novel therapeutic strategy for NSCLC. In order to explore the important roles of microRNAs playing in early NSCLC, we initially searched for a group of data from the GEO database (GSE42425), which included 71 NSCLC lung tissues of stage I, and compared their microRNA expressions.

**Figure 1:**
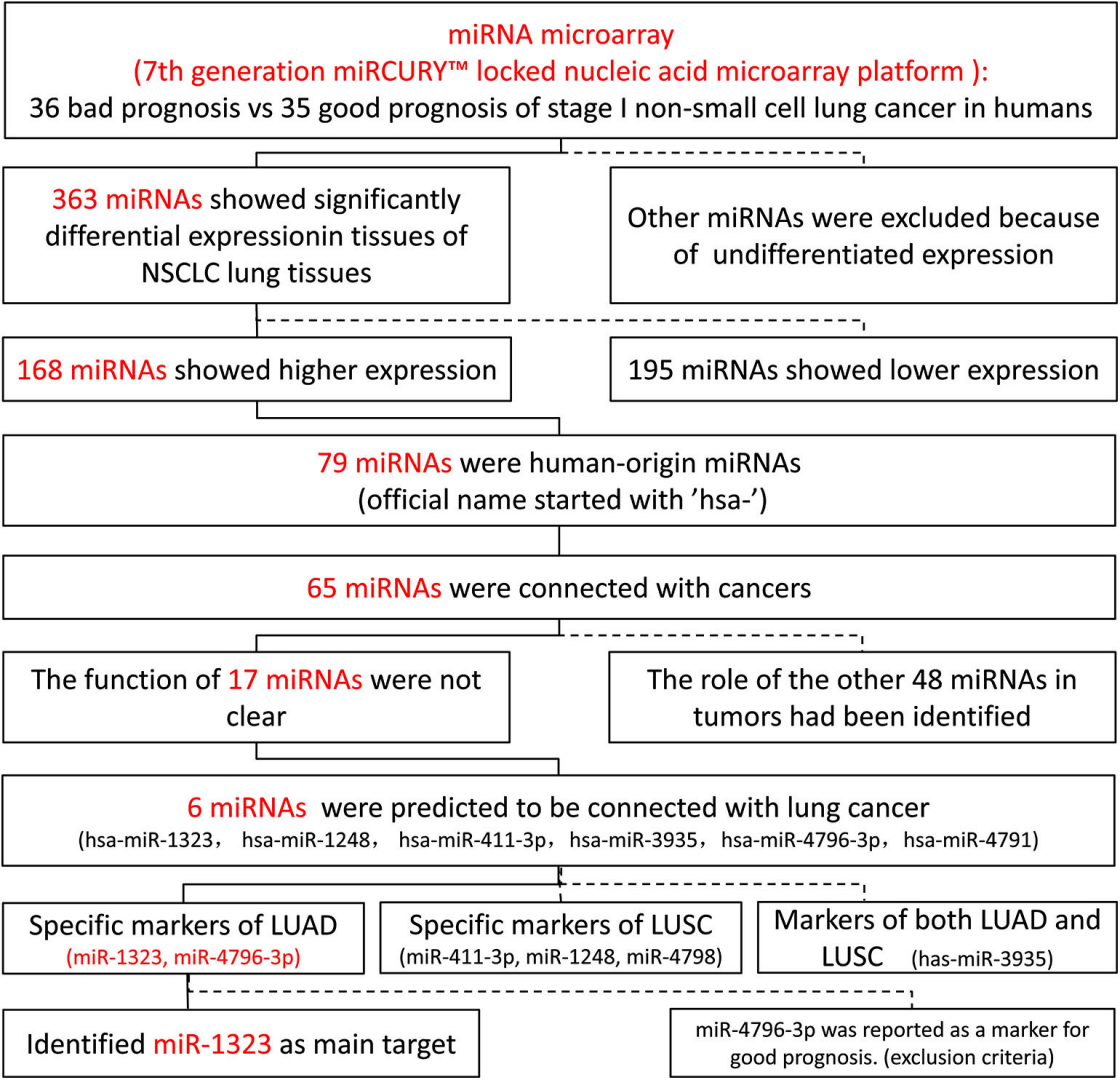
Screening process of vital microRNA of early NSCLC.

**Figure 2:**
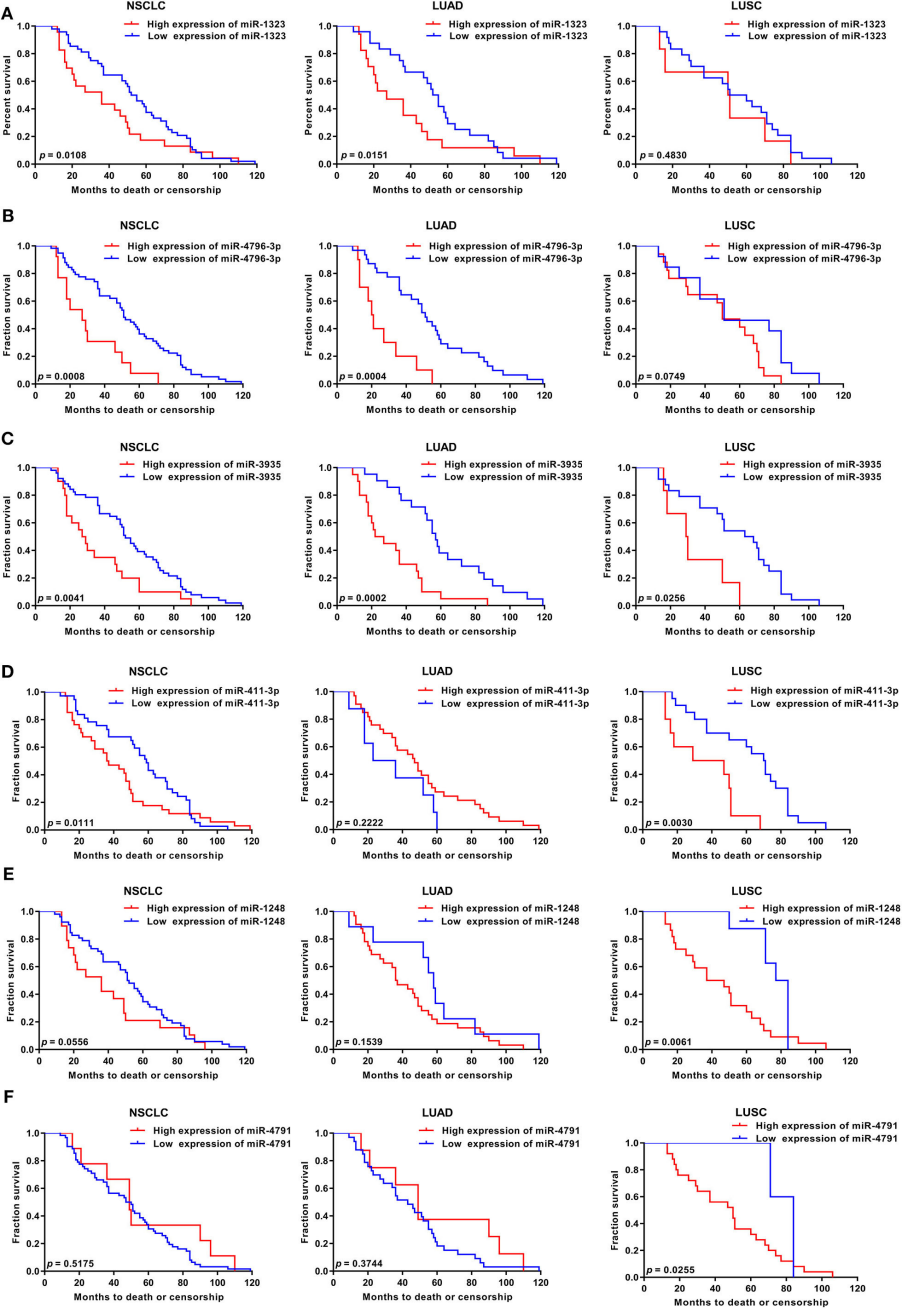
KM survival curve and log-rank test for patients with six miRNAs high xpression in NSCLC, LUAD, and LUSC using GEO database. Using GSE42425 from GEO database, the expression of **(A)** miR-1323, **(B)** miR-4796-3p, **(C)** miR-3935, **(D)** miR-411-3p, **(E)** miR-1248, **(F)** miR-4791, and with OS in NSCLC, LUAD, and LUSC.

As shown in Figure 1, 363 miRNAs were found to be differently expressed in patients with short overall survival than in those with long overall survival according to the criteria of fold change >1.2. Among these miRNAs, 168 showed higher expression in NSCLC patients with poor prognosis, and 79 miRNAs were chosen as they were human-origin miRNA. Afterwards, we searched the literature and screened out 65 miRNAs that had been identified their potential crucial roles in tumors. Since we hoped to find the key miRNA that could serve as clinical markers or targets for early diagnosis in NSCLC, 48 miRNAs were excluded owing to the weaker relationship with cancer. Among the remaining 17 miRNAs, only six were reported to be associated with lung cancer. During the process of analysis of these six miRNAs, we surprisingly found that they caused different effects to prognosis according to varies of pathological types. Two miRNAs (miR-1323, miR-4796) might be the alternative marker for LUAD (Figures 2A,B), while one miRNA (miR-3935, Figure 2C) is pathological type non-specific indication andthree miRNAs (miR-411-3p, miR-1248, miR-4791) have a clear indication for prognosis of lung squamous cell carcinoma (LUSC, Figures 2D–F). Since miR-4796 was reported that its low expression was associated with primary resistance of EGFR-tyrosine kinase inhibitors (EGFR-TKIs), which is the first-line treatment of LUAD. Furthermore, little literature had concerned about it. Thus, we chose miR-1323 for further experiments. The characteristics of the patients in GSE42425 are shown in Table 1. We found that there was no significant association between the expression levels of miR-1323 and other clinicopathological parameters except histological type, which predicts that miR-1323 has important prognostic significance in lung adenocarcinoma.

**Table 1:** Characteristics of patients in GSE42425.

**Characteristic**	**Number**	**miR-1323 expression**		***P*-value**
		**High**	**Low**	
**Age, years**	68 (46.0–83.0)			0.614
Median (range)				
≤68.0	36 (50.7)	23 (47.9)	13 (56.5)	
>68.0	35 (49.3)	25 (52.1)	10 (43.5)	
**Gender, no. (%)**				0.607
Male	34 (47.9)	24 (50.0)	10 (43.5)	
Female	37 (52.1)	24 (50.0)	13 (56.5)	
**Histological type, no (%)**				0.056
LUAD	41 (40.6)	24 (50.0)	17 (73.9)	
LSCC	30 (39.1)	24 (50.0)	6 (26.1)	
**Race, no (%)**				0.677
White	65 (91.5)	44 (91.7)	21 (91.3)	
Black	4 (6.3)	2 (4.3)	2 (8.7)	
Asian	1 (1.6)	1 (2.0)	0 (0.0)	
American indian	1 (1.6)	1 (2.0)	0 (0.0)	
**History of smoking, no (%)**				0.929
Never	8 (11.3)	5 (10.4)	3 (13.0)	
Former	43 (60.6)	29 (60.4)	14 (60.8)	
Current	20 (28.2)	14 (29.2)	6 (26.1)	
**Stage, no (%)**				0.613
IA	38 (53.5)	27 (56.3)	11 (47.8)	
IB	33 (46.5)	21 (43.7)	12 (52.2)	
**Status of recurrence of cancer**				0.405
No	39 (54.9)	28 (58.3)	11 (47.8)	
Yes	32 (45.1)	20 (41.7)	12 (52.2)	

*LUAD, lung adenocarcinoma; LUSC, Lung squamous cell carcinoma; BAC, bronchioloalveolar carcinoma; AS, adenosquamous carcinoma*.

### Validation the Effect of miR-1323 in an Independent Cohort of LUAD Patients

**Figure 3:**
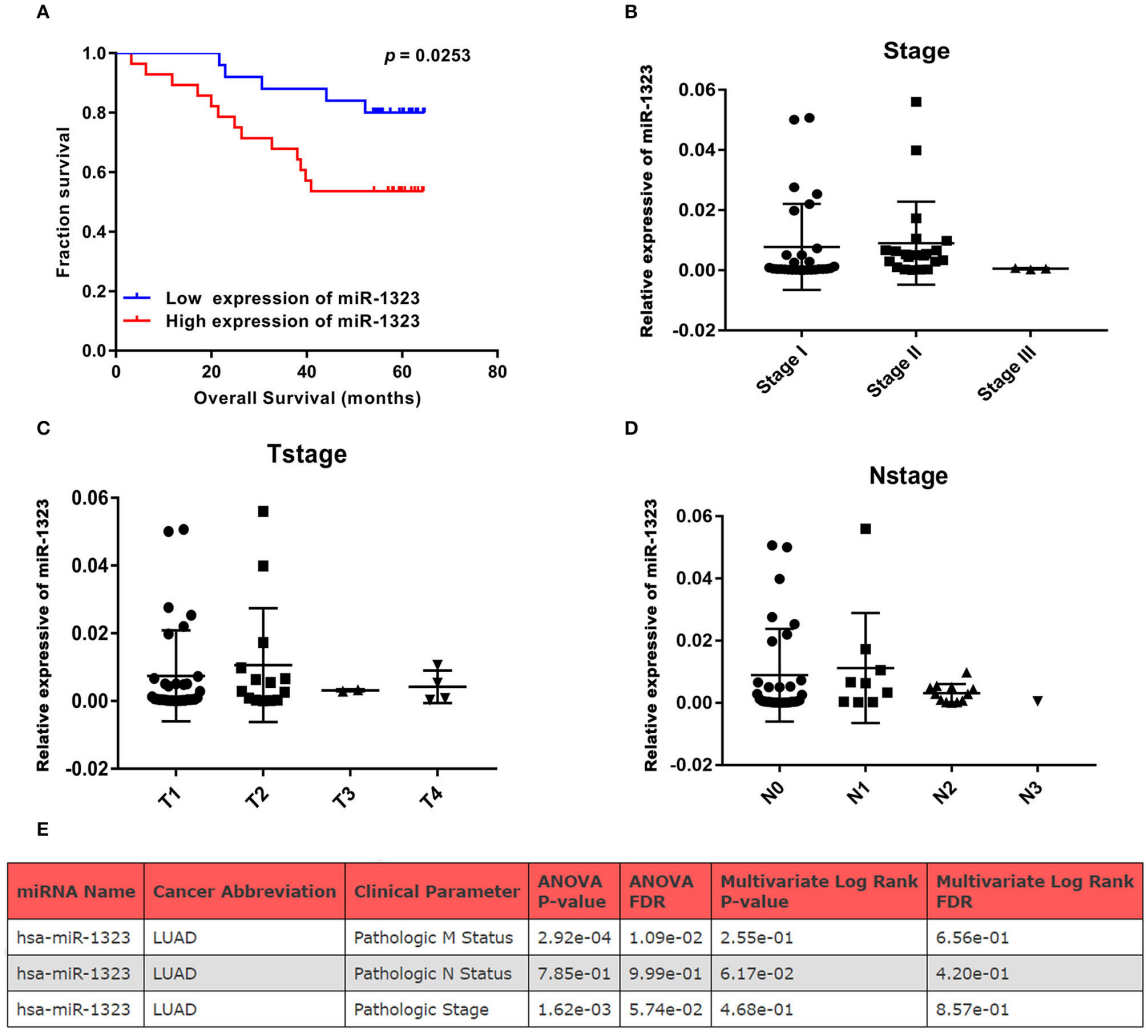
KM survival curve and pathological stage for miR-1323 expression in LUAD cases. **(A)** KM survival curve of OS for 54 LUAD cases. **(B)** Pathological stage, **(C)** T stage, **(D)** N stage of 54 LUAD cases. **(E)** The relation between clinical parameters in LUAD and hsa-miR-1323 by ONCOMIR.

In order to verify the prognosis predictive function of miR-1323 expression in LUAD, we selected 53 LUAD cases that underwent resection at Shengjing Hospital of China Medical University (Table 2). The cohort, analyzed for the effect of prognosis, included 53 patients (30 men, 23 women) with a median age at surgery of 59 years (range, 35–75 years). Of these, 37 (69.8%) patients were at clinical stage I or II, and 16 (30.2%) were at stage III. The median survival duration was 57 months. Using surgically resected and paraffin sections from these cases, we performed qRT-PCR, and found that the expression level of miR-1323 in the paraffin sections ranged from 0.000085 to 0.056. Using the X-tile program, we chose an optimal cut-off value to divide the patients into two groups (miR-1323 low expression and miR-1323 high expression). Kaplan Meier survival analysis results showed high miR-1323 expression was found to indicate poor prognosis in LUAD (*P* = 0.0253, Figure 3A). In detail, higher expression of miR-1323 was associated with shorter overall survival (OS) in NSCLC and LUAD. These suggest that miR-1323 would like to be used as a specific early prognostic marker for LUAD to guide clinical treatment.

**Table 2:** Correlation of clinical features of NSCLC samples with miR-1323 expression levels of NSCLC cases.

**Characteristic**	**Number**	**miR-1323 expression**		***P***
		**High expression**	**Low expression**	
**Age, years, no. (%)**				0.884
Median (range)	59.0 (35.0–75.0)			
≤59.0	27 (50.9)	13 (52.0)	14 (50.0)	
>59.0	26 (49.1)	12 (48.0)	14 (50.0)	
**Gender, no. (%)**				0.033*
Male	30 (56.6)	12 (42.9)	18 (72.0)	
Female	23 (43.4)	16 (57.1)	7 (28.0)	
**EGFR mutant state, no. (%)**				0.674
19-Del	15 (28.3)	7 (25.0)	8 (32.0)	
L858R	18 (34.0)	11 (39.3)	7 (28.0)	
Unknown	20 (37.7)	10 (35.7)	10 (40.0)	
**Depth of invasion, no (%)**				0.471
T1+T2	47 (88.7)	24 (85.7)	23 (92.0)	
T3+T4	6 (11.3)	4 (14.3)	2 (8.0)	
**Lymph node status, no (%)**				0.442
N0–1	31 (58.5)	15 (53.6)	16 (64.0)	
N2–3	22 (41.5)	13 (46.4)	9 (36.0)	
**TNM Stage, no (%)**				0.354
I+II	37 (69.8)	18 (64.3)	19 (76.0)	
III	16 (30.2)	10 (35.7)	6 (24.0)	

**p < 0.05.

*LUAD, lung adenocarcinoma; LUSC, Lung squamous cell carcinoma; BAC, bronchioloalveolar carcinoma; AS, adenosquamous carcinoma*.

Cox univariate and multivariate regression analysis was performed on the prognostic value of pathological parameters such as age, gender, T stage, N stage, M stage, and miR-1323 expression using the above data. Univariate analysis showed that T-stage, N-stage, and miR-1323 expression levels were prognostic factors for lung adenocarcinoma (*P* < 0.1, Table 3). Further applying of Cox regression model, further multivariate analysis of factors with *P* < 0.1 in univariate analysis showed that N-stage and miR-1323 expression in tumor patients were independent prognostic factors for lung adenocarcinoma. These results suggest that miR-1323 is an independent risk factor for the prognosis of lung adenocarcinoma.These data further vertified the important role of mir-1323 in guiding prognosis for LUAD patients.

**Table 3:** Univariate and multivariate analysis of the influence of clinicopathological features on prognosis in lung adenocarcinoma.

**Variables**	**Univariate analysis Multivariate analysis**					
	**HR**	**95% CI**	***P***	**HR**	**95% CI**	***P***
Age (years)	0.985	0.941–1.032	0.530			
Gender (male vs. female)	1.548	0.580–4.126	0.383			
T staging	2.560	0.840–7.803	0.098^*^			
N staging	2.595	1.005–6.701	0.049^**^	2.541	0.983–6.567	0.054^*^
EGFR mutant state	1.044	0.732–1.489	0.811			
mir-1323	2.937	1.045–8.255	0.041^**^	2.881	1.024–8.108	0.045^**^

** *p < 0.05,*

* *p < 0.1*.

### miR-1323 Promotes Migration of A549 and HCC827 Cells

**Figure 4:**
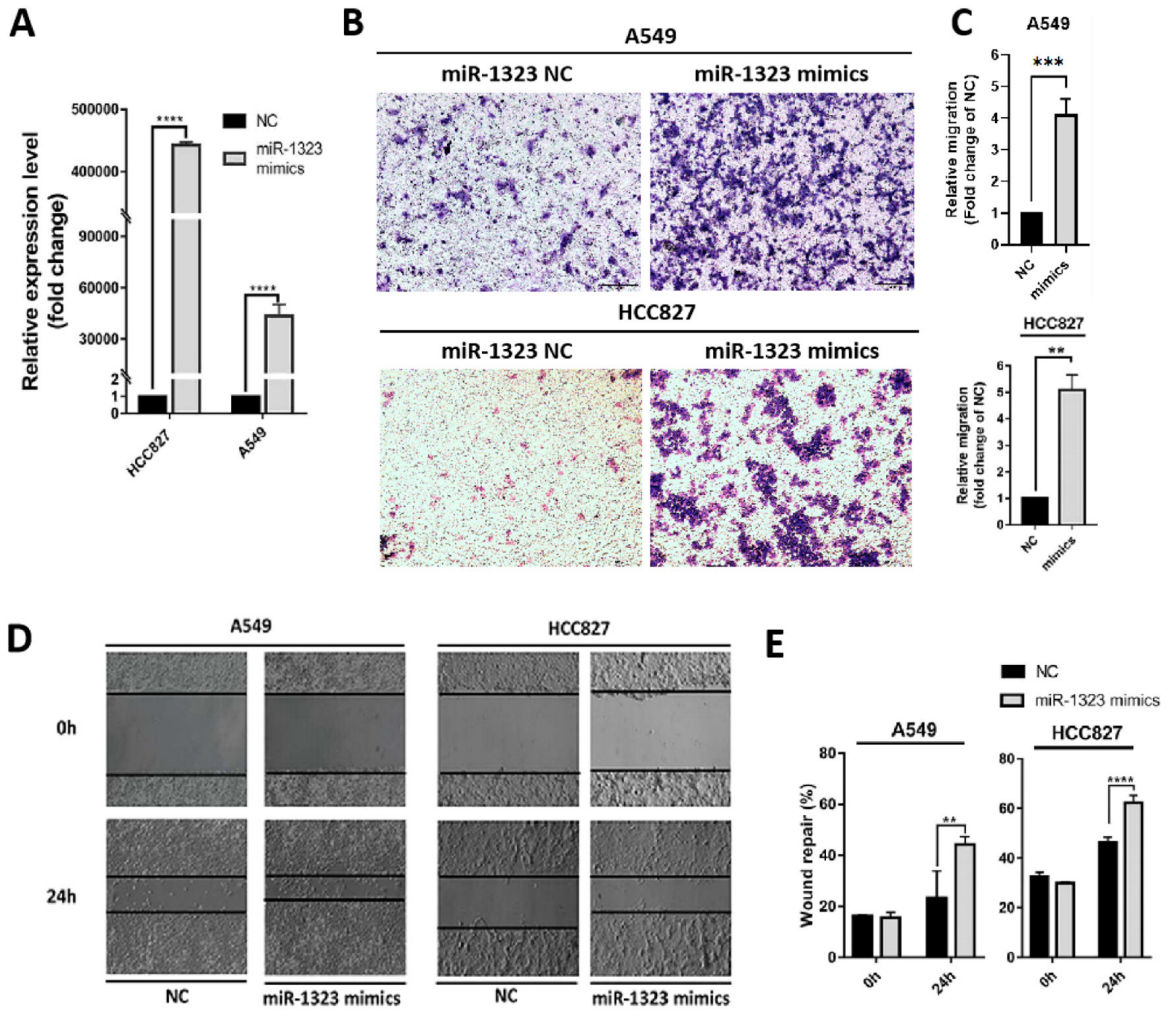
Overexpression of miR-1323 significantly promotes the migration of HCC827cells and A549 cells. **(A)** A549 cells and HCC827 cells were transfected with miR-1323 mimics or NC, qRT-PCR was used to confirm the overexpression efficiency of miR-1323 mimics. **(B,C)** Transwell assays and **(D,E)** wound healing assays was to detect the migration of A549 cells and HCC827 cells. *t*-test was used to assess statistically significant differences between groups. Mean ± SD, results of three independent experiments, **p < 0.01, ****p < 0.0001.

Considering that mir-1323 has such a conductive effect on the prognosis of LUAD patients, we wonder that how it participates in regulating biological behavior and if it could be a novel target for clinical treatment. OncomiR, an online resource for exploring miRNA dysregulation in cancer, indicated that mir-1323 expression was correlated with pathologic M Status and N status (Figure 3E). Considering the Kaplan–Meier survival analysis showed that LUAD patients with high miR-1323 expression had shorter overall survival shown in Figure 2, we speculated higher miR-1323 expression might imply higher migration ability for LUAD. In order to investigate the effect of miR-1323 on LUAD, lung cancer cell lines A549 and HCC827 were transfected with an miR-1323 mimics or a negative control (NC) for 24 h (Figure 4A). The experimental results of both transwell assays and wound healing assays showed that miR-1323 significantly increased the migration of A549 cells and HCC827 cells post-transfection compared to the negative control groups (Figures 4B–E), which indicated the way of miR-1323 leading poor prognosis of LUAD patients was by promoting tumor cell metastasis.

### Cbl-b Is the Target of miR-1323 in NSCLC and Related With Patients' Prognosis

**Figure 5:**
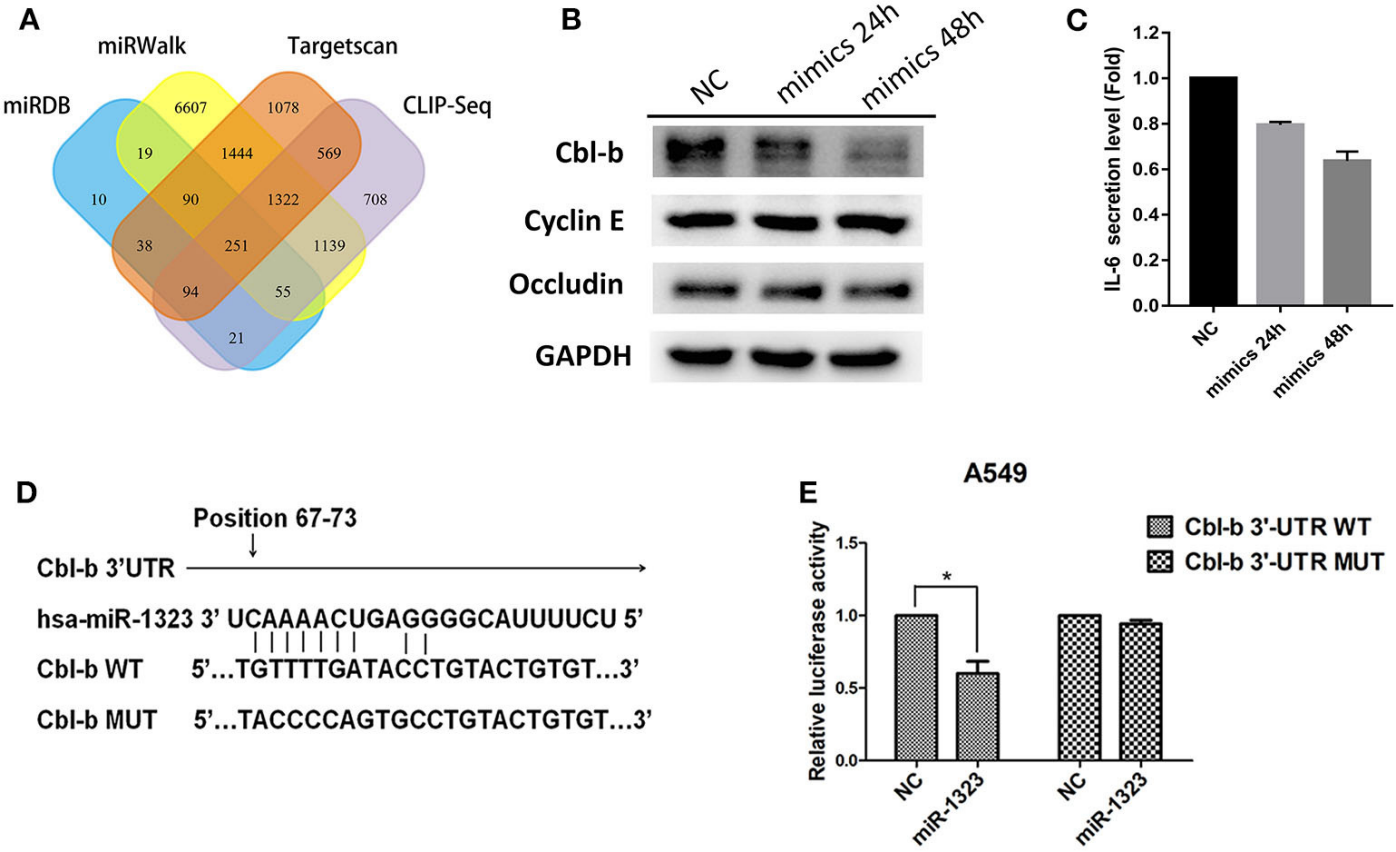
miR-1323 can promotes the migration of A549 cells through inhibiting Cbl-b. **(A)** A schematic view of miR-1323 predicted target genes through four miRNA databases of different mechanisms. The A549 cells were transfected with miR-1323 mimic or NC, **(B)** WB detected the expression of proteins and **(C)** Elisa detected the secretory level of IL-6 in A549 cells. **(D)** The bindng site of miR-1323 to Cbl-b 3′UTR. **(E)** The A549 cells were co-transfected with pMirTarget-Cbl-b WT (or MUT) plasmid, miR-1323 mimic (or NC), and pRL-TK. The dual luciferase reporter assay detected the activity of luciferase. The histogram shows the relative activity of luciferase. *t*-test was used to assess statistically signifificant differences between groups. Mean ± SD, results of three independent experiments, *p < 0.05.

To examine the mechanisms through which miR-1323 exerts its lung cancer-promoting effects, we used bioinformatic algorithms (Figure 5A) and identified 251 potential target genes of miR-1323, among which only four genes are common tumor- associated genes related with prognosis of patients with lung adenocarcinoma in the Kaplan- Meier Plotter database (*P* < 0.05). After miR-1323 overexpression in A549 cells, only the protein expression level of Cbl-b was obviously suppressed among these candidates (Figure 5B). The level of IL-6 was generally measured by ELISA (Figure 5C). Since the expression level of IL-6 was negatively correlated with miR-1323 mimics in a time-dependent manner in A549 cells, which is inconsistent with the fact that IL-6 promotes inflammation and promotes cancer as it was reported, it wouldn't be considered as the main target gene of miR-1323 in the development of lung adenocarcinoma. Additionally, using computational algorithms, we identified that the 3′UTR of Cbl-b mRNA contains a complementary binding site for the miR-1323 seed region (Figure 5D). We also performed 3′UTR luciferase reporter assays to validate whether miR-1323 directly targets Cbl-b. Cotransfection of miR-1323 with the wild type 3′UTR of Cbl-b significantly repressed the relative luciferase (*P* < 0.05), and cotransfection of miR-1323 with the mutated 3′UTR of Cbl-b has no effects (*P* = 0.100), suggesting that miR-1323 targets Cbl-b directly (Figure 5E). These results indicate that Cbl-b is a target gene of miR-1323.

### miR-1323 Promotes the Migration of NSCLC Cells Through Inhibiting Cbl-b

**Figure 6:**
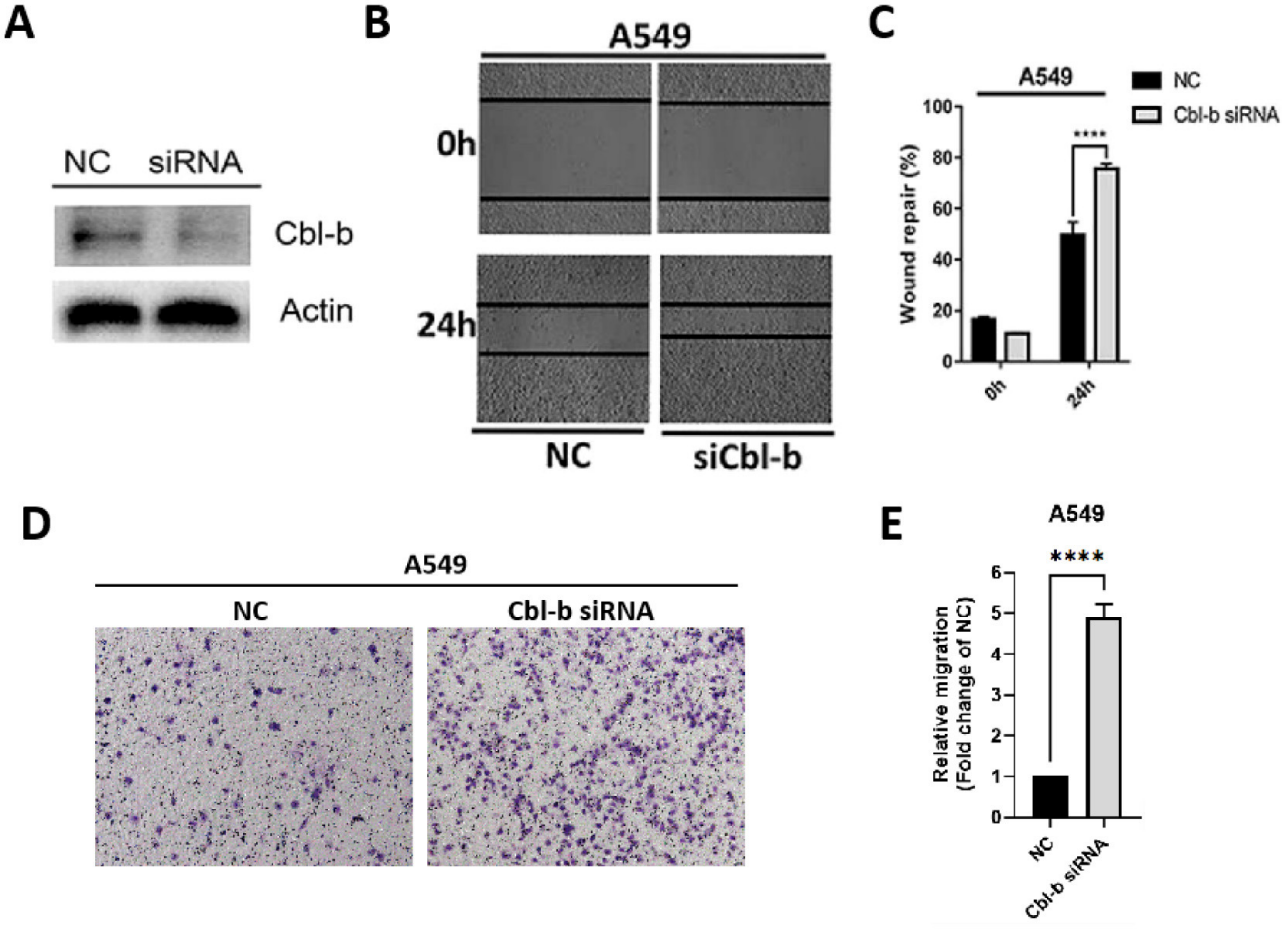
Decreasing the expression of Cbl-b increases the migration ability of A549 cells. A549 cells were transfected with Cbl-b siRNA or NC, **(A)** WB detected the expression of Cbl-b in A549 cells. **(B,C)** Wound healing assays and **(D,E)** transwell assays showed the migration ability of A549 cells. *t*-test was used to assess statistically signifificant differences between groups. Mean ± SD, results of three independent experiments, *p < 0.05, ****p < 0.0001.

**Figure 7:**
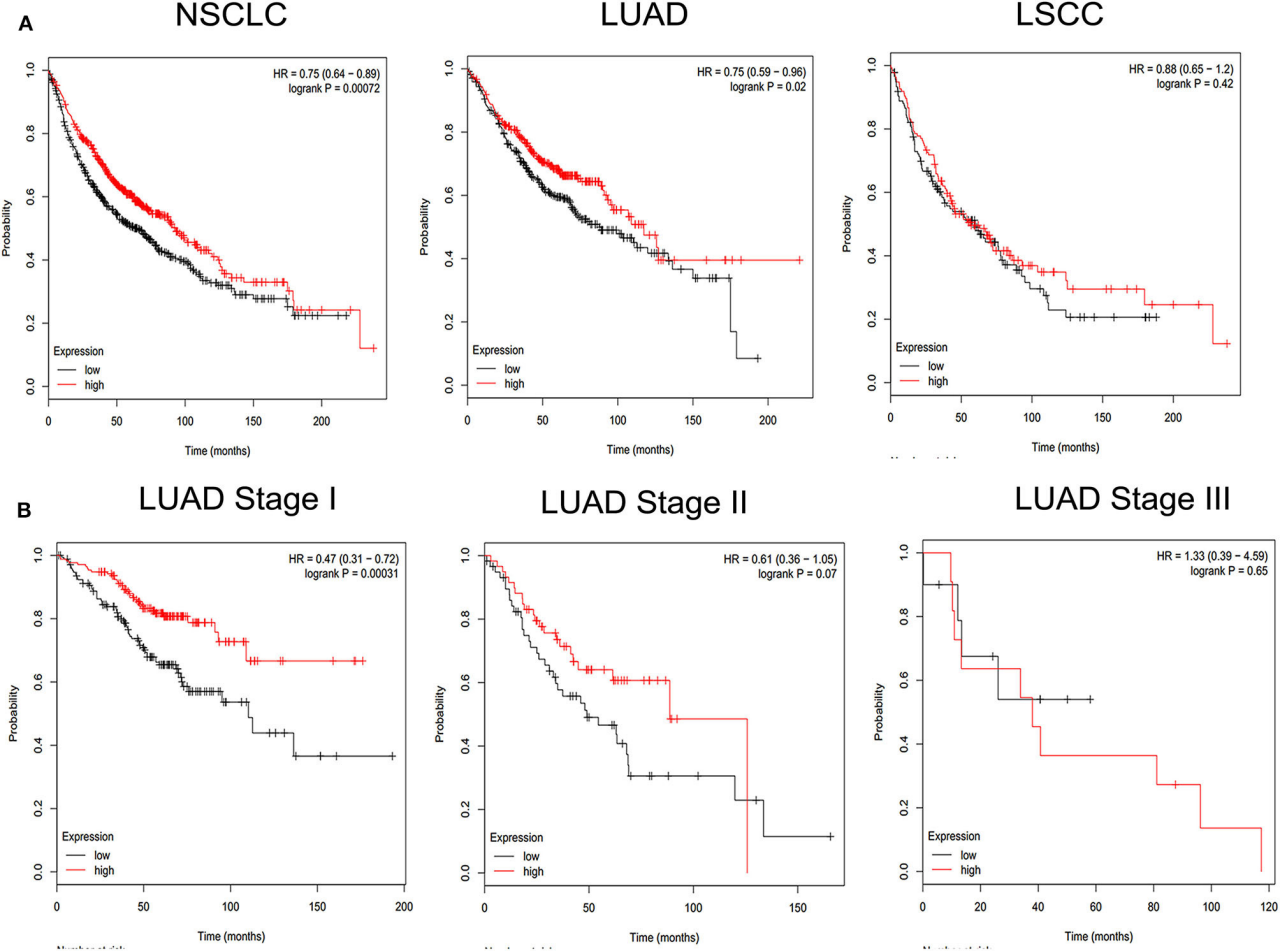
KM survival curve and log-rank test for patients with Cbl-b high or low expression in different pathological types of NSCLC and LUAD at Stage I, Stage II, Stage III. Online Kaplan Meier plotter Database was used to analysis. **(A)** KM survival curve of Cbl-b in non-small cell lung cancer (NSCLC), lung adenocarcinoma (LUAD), lung squamous carcinoma (LUSC). **(B)** KM survival curve of Cbl-b in different pathological stage in LUAD.

To investigate the detailed relationship between miR-1323 and Cbl-b and the effect of Cbl-b on the migration in NSCLC cells, we further transfect Cbl-b siRNA and a negtive control in A549 and HCC827 cells. The transfection efficiency was ensured by western blot analysis (Figure 6A). Treatment with Cbl-b siRNA significantly promoted the cell migration when compared to that with the negtive control in both A549 cells (Figures 6B–E). These results emphasize miR-1323 inhibits the expression of Cbl-b and promotes the metastasis of tumor cells, leading to poor prognosis in patients with early LUAD. Besides, we used the Kaplan-Meier plotter and found that Cbl-b overexpression was associated with a long overall survival time in NSCLC (*P* = 0.00072) and in LUAD (*P* = 0.02,), but there is not significant differences in LUSC (Figure 7). Additionally, high Cbl-b expression was significantly associated with better prognosis in stage I (*P* = 0.00031), whereas there was no significant association in stage II and III (*P* = 0.07 and *P* = 0.65, Figure 7). To summarize, Cbl-b, whose low expression indicates poor prognosis in stage I, is the target of miR-1323 of NSCLC patients and the correlation between Cbl-b and prognosis depends on histological type and clinical stage.

## Discussion

Previous studies have shown that microRNAs participate in lung cancer development and progression, and may be potential prognostic biomarkers for lung cancer (19, 20). In this study, miR-1323 was selected through an online database, and was found to predict the survival of lung cancer patients. We validated miR-1323 in an independent cohort and demonstrated that high miR-1323 expression predicted poor survival in LUAD. Mechanistically, miR-1323 promoted the migration of lung cancer cells by targeting Cbl-b.

It is well-known that microRNAs are involved in multiple biological processes such as cell differentiation, proliferation, apoptosis, EMT, and cell migration, through targeting gene expression (21–23). Previously, a study has shown that miR-1323 promoted radiation-resistant lung cancer cells (10). However, the effect of miR-1323 in LUAD is unknown. In the present study, we examined the expression of miR-1323 in 53 LUAD, and found that its high expression predicted poor survival in LUAD (*P* = 0.0253). It is worth noting that the data from the GEO database (GSE42425) came from different populations, including the Whites, Blacks, Asians, and American Indians, but mainly the Whites. The patients' sample data from our results is mainly the Asians. However, both cohorts show that the expression of miR-1323 affects the prognosis of patients with LUAD. These results validated miR-1323 as a potential prognostic marker in LUAD patients.

The executive effect of miR-1323 in LUAD is still unknown. In the present study, we found that miR-1323 promoted cell migration in LUAD cell lines A549 and HCC827. A previous study has shown that PRKDC was the target gene of miR-1323, which was involved in radiation resistance.In the present study, we used bioinformatics tools to predict the potential target genes of miR-1323 and found Cbl-b as a potential target gene, which was validated by the dual luciferase reporter gene assay. Cbl-b was found to be a direct target gene of miR-1323. Cbl-b is the second member of the c-Cbl RING finger E3 ubiquitin protein ligases. It targets tyrosine-kinase receptors and growth signaling proteins for ubiquitination and down regulation (24–26). Our previous studies demonstrated that Cbl-b inhibited migration of gastric cancer and breast cancer cell (27, 28). However, whether Cbl-b is involved in the migration of lung cancer cells was unknown. In the present study, we first demonstrated Cbl-b was the target gene of miR-1323, and inhibited the migration of A549 cells. Additionally, analysis of an online database showed that Cbl-b is associated with the survival of LUAD patients in stage I.

In conclusion, our present study highlights the important significance of miR-1323 in predicting the poor survival in early LUAD and regulating of lung adenocarcinoma cells metastasis by targeting Cbl-b. We provide a potential early diagnosis biomarker for lung adenocarcinoma, which could help lung cancer patients development effective and appropriate strategies as early as possible when transferred into clinical practice. However, the mechanism of miR-1323 needs to be further explored. Our team will devote ourselves to in-depth research based on present results.

## Data Availability Statement

Publicly available datasets were analyzed in this study. This data can be found here: https://www.ncbi.nlm.nih.gov/geo/query/acc.cgi?acc=GSE42425. The datasets generated for this study are available on request to the corresponding author.

## Ethics Statement

The studies involving human participants were reviewed and approved by the Research Ethics Committee of Shengjing Hospital of China Medical University and all patients signed informed consent forms to allow analyses to be performed on their tissue samples.

## Author Contributions

HZ, XH, and YF contributed conception and design of the study. DP, CZ, and KH gave the technical support. XY, SX, SW, and TZ performed the experiment and statistical analysis. YW, XC, and YC wrote the manuscript. JK, YL, XH, and XQ provided fund supports. All authors contributed to manuscript revision, read, and approved the submitted version.

### Conflict of Interest

The authors declare that the research was conducted in the absence of any commercial or financial relationships that could be construed as a potential conflict of interest.

## Correction note

A correction has been made to this article. Details can be found at: 10.3389/fonc.2026.1904016.
