# Correction: miR-1323 promotes cell migration in lung adenocarcinoma by targeting Cbl-b and is an early prognostic biomarker

**DOI:** 10.3389/fonc.2026.1904016

**Published:** 2026-07-03

**Authors:** Huan Zhao, Chunlei Zheng, Yizhe Wang, Kezuo Hou, Xianghong Yang, Yang Cheng, Xiaofang Che, Shilin Xie, Shuo Wang, Tieqiong Zhang, Jian Kang, Yunpeng Liu, Dianzhu Pan, Xiujuan Qu, Xuejun Hu, Yibo Fan

**Affiliations:** 1 Department of Respiratory and Infectious Disease of Geriatrics, The First Hospital of China Medical University, Shenyang, China; 2 Department of Respiratory, The First Affiliated Hospital of Jinzhou Medical University, Jinzhou, China; 3 Department of Medical Oncology, The First Hospital of China Medical University, Shenyang, China; 4 Key Laboratory of Anticancer Drugs and Biotherapy of Liaoning Province, The First Hospital of China Medical University, Shenyang, China; 5 Department of Pathology, Shengjing Hospital of China Medical University, Shenyang, China; 6 Department of Pulmonary Medicine, The First Hospital of China Medical University, Shenyang, China

**Keywords:** lung adenocarcinoma, miR-1323, CBLB, prognosis, biomarker

The reference for “6. Xu X, Cao L, Zhang Y, Lian H, Sun Z, Cui Y. MicroRNA-1246 inhibits cell invasion and epithelial mesenchymal transition process by targeting CXCR4 in lung cancer cells. Cancer Biomark. (2018) 21:251–60. 10.3233/CBM-170317” was retracted. It should be replaced by “6.Wang L, Qu J, Zhou L, Liao F, Wang J. MicroRNA-373 Inhibits Cell Proliferation and Invasion via Targeting BRF2 in Human Non-small Cell Lung Cancer A549 Cell Line. Cancer Res Treat. (2018) 50(3):936-949. doi: 10.4143/crt.2017.302”.

The reference for “7. Qian L, Ji AH, Zhang WJ, Zhao N. HuR, TTP, and miR-133b expression in NSCLC and their association with prognosis. Eur Rev Med Pharmacol Sci. (2018) 22:430–42. 10.26355/eurrev_201801_14192” was retracted. It should be replaced by “7. Chen GY, Ruan L. Downregulation Of microRNA-133b And Its Clinical Value In Non-Small Cell Lung Cancer. Onco Targets Ther. (2019) 12:9421-9434. doi: 10.2147/OTT.S231312”.

The reference for “21. Jiang J, Yi B, Qin C, Ding S, Cao W. Upregulation of microRNA27b contributes to the migration and invasion of gastric cancer cells via the inhibition of sprouty2mediated ERK signaling. Mol Med Rep. (2016) 13:2267–72. 10.3892/mmr.2016.4779” was retracted. It should be replaced by “21. Peng Y, Croce CM. The role of MicroRNAs in human cancer. Signal Transduct Target Ther. (2016)1:15004. doi: 10.1038/sigtrans.2015.4”.

There was a mistake in **Figure 4B** as published. The image of migration of miR-1323 mimics in A549 cells (the second image on the first line) in **Figure 4B** was repeated. The corrected **Figure 4** appears below.

**Figure 4:**
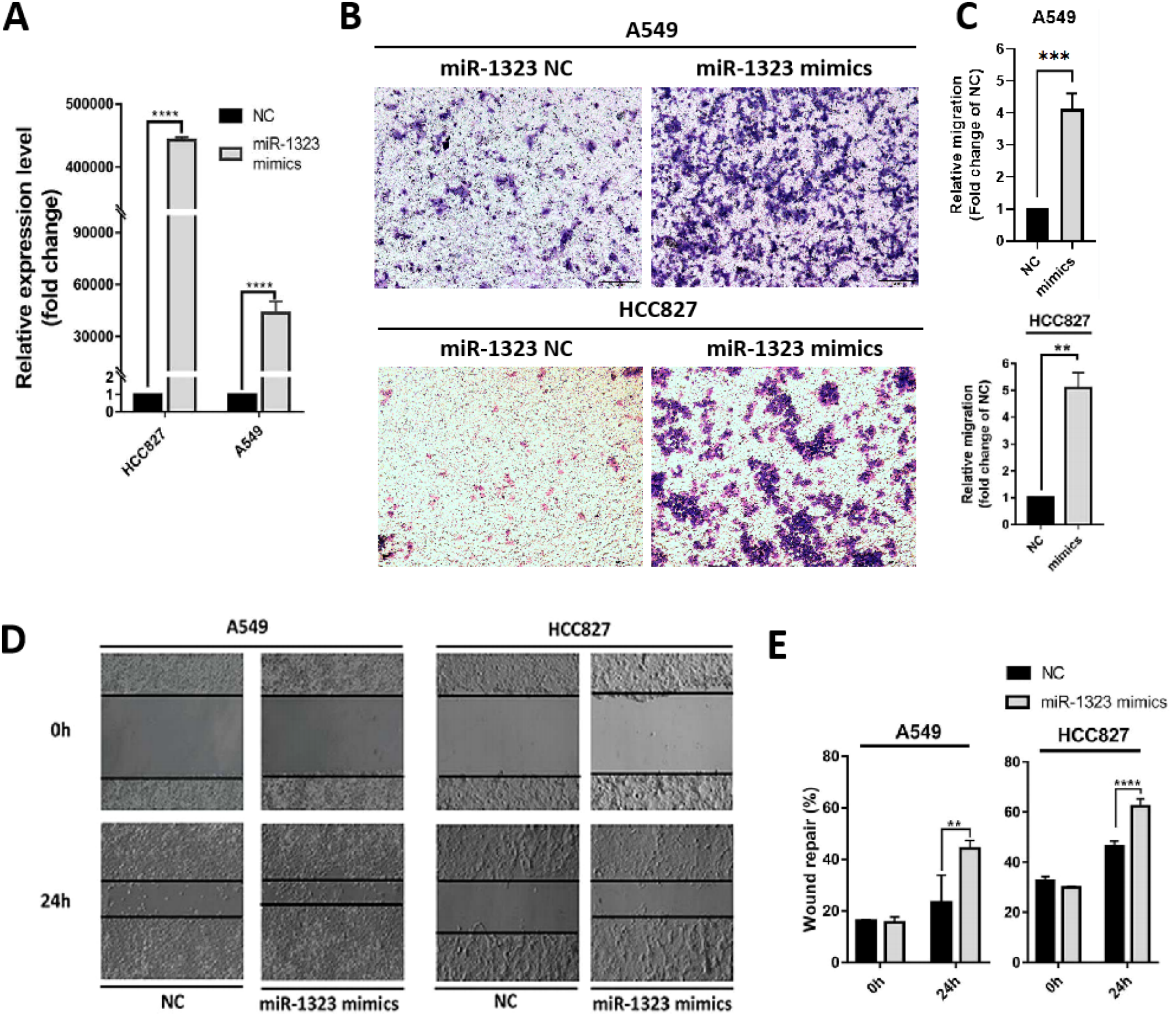
Overexpression of miR-1323 significantly promotes the migration of HCC827cells and A549 cells. **(A)** A549 cells and HCC827 cells were transfected with miR-1323 mimics or NC, qRT-PCR was used to confirm the overexpression efficiency of miR-1323 mimics. **(B, C)** Transwell assays and **(D, E)** wound healing assays was to detect the migration of A549 cells and HCC827 cells. t-test was used to assess statistically significant differences between groups. Mean ± SD, results of three independent experiments, **p < 0.01, ****p < 0.0001.

There was a mistake in **Figure 6D** as published. The image of migration of Cbl-b siRNA in A549 cells (the second image) in **Figure 6D** was repeated. The corrected **Figure 6** appears below.

**Figure 6:**
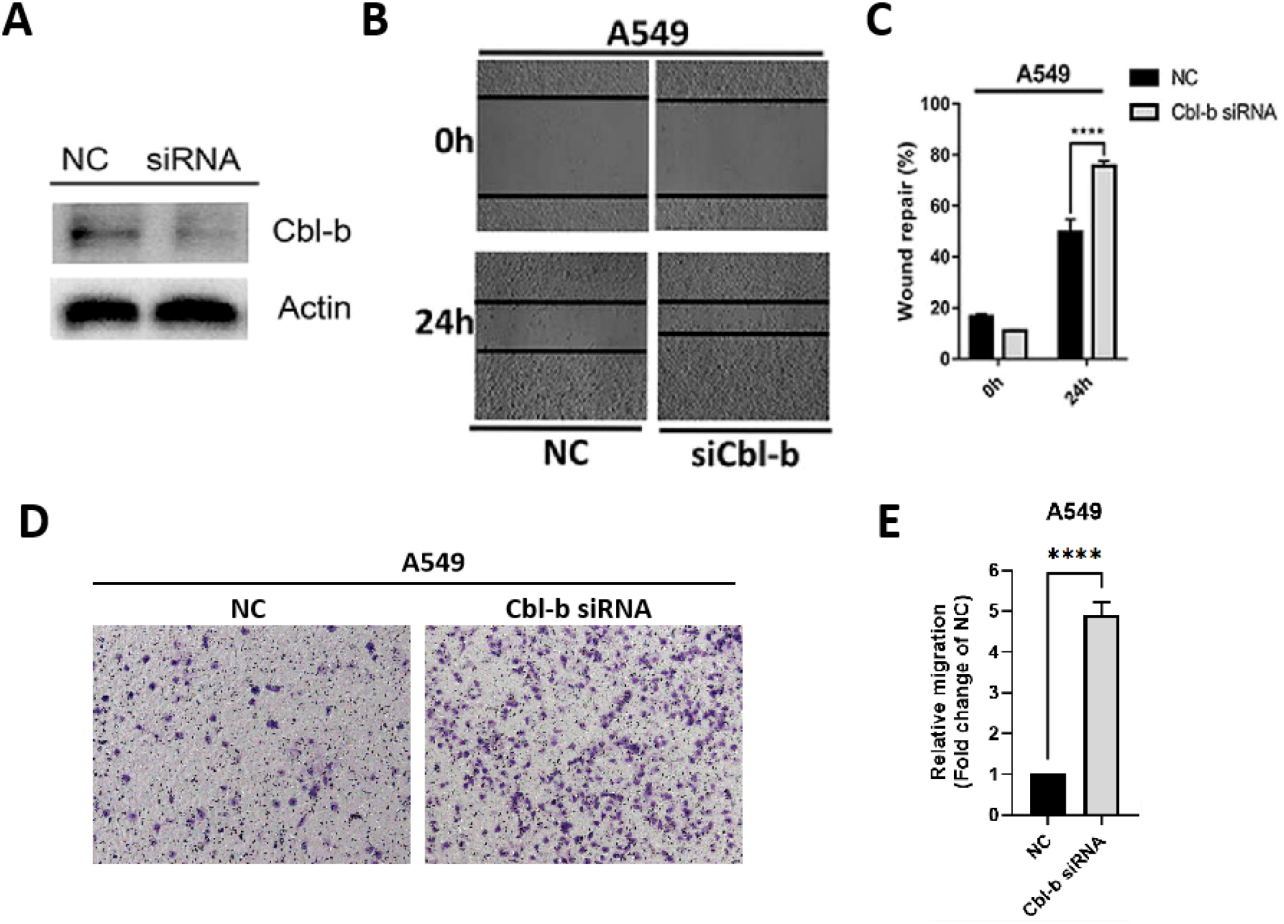
Decreasing the expression of Cbl-b increases the migration ability of A549 cells. A549 cells were transfected with Cbl-b siRNA or NC, **(A)** WB detected the expression of Cbl-b in A549 cells. **(B, C)** Wound healing assays and **(D, E)** transwell assays showed the migration ability of A549 cells. t-test was used to assess statistically signifificant differences between groups.Mean ± SD, results of three independent experiments, *p < 0.05, ****p < 0.0001.

The original article has been updated.

